# Applying models of self-regulated learning to understand engagement with digital health interventions: a narrative review

**DOI:** 10.3389/fdgth.2025.1380088

**Published:** 2025-05-09

**Authors:** Claudia Liu, Mariel Messer, Jake Linardon, Matthew Fuller-Tyszkiewicz

**Affiliations:** ^1^ School of Psychology, Deakin University, Melbourne, VIC, Australia; ^2^ Centre for Social and Early Emotional Development, Deakin University, Burwood, VIC, Australia

**Keywords:** digital health interventions, user engagement, self-regulated learning, narrative review, digital health

## Abstract

Digital health interventions (DHIs) are often burdened by poor user engagement and high drop-out rates, diminishing their potential public health impact. Identifying user-related factors predictive of engagement has therefore drawn significant research attention in recent years. Absent from this literature—yet implied by DHI design—is the notion that individuals who use DHIs have well-regulated learning capabilities that facilitate engagement with unguided intervention content. In this narrative review, we make the case that learning capacity can differ markedly across individuals, and that the requirements of self-guided learning for many DHIs do not guarantee that those who sign up for these interventions have good learning capabilities at the time of uptake. Drawing upon a rich body of theoretical work on self-regulated learning (SRL) in education research, we propose a user-as-learner perspective to delineate parameters and drivers of variable engagement with DHIs. Five prominent theoretical models of SRL were wholistically evaluated according to their relevance for digital health. Three key themes were drawn and applied to extend our current understanding of engagement with DHIs: (a) common drivers of engagement in SRL, (b) the temporal nature of engagement and its drivers, and (c) individuals may differ in learning capability. Integrating new perspectives from SRL models offered useful theoretical insights that could be leveraged to enhance engagement with intervention content throughout the DHI user journey. In an attempt to consolidate these differing—albeit complementary—perspectives, we develop an integrated model of engagement and provide an outline of future directions for research to extend the current understanding of engagement issues in self-guided DHIs.

## 1 Introduction

Digital health interventions (DHIs) are self-guided therapeutic mental health interventions electronically administered through devices such as smartphones, tablets, and computers (1). Common forms of DHIs include app-based interventions—where the therapeutic program is delivered using a mobile application, and web-based interventions—that are delivered via an online web browser on a computer. With the growing reach of the internet and a global estimate of almost 6.9 billion smartphone users in 2023 (2), there is promise for digital health to be a widely accessible and scalable component of 21st century healthcare systems alongside traditional, physically-located services such as hospitals and clinics. DHIs are viewed favorably because of their potential to overcome a number of barriers associated with face-to-face treatments including geographical constraints, shortages of trained professionals, desire for anonymity, and out-of-pocket expenses (3). Other potentially desirable features include their ability to monitor symptoms over time and to offer real-time symptom relief at pivotal life moments (1).

Despite these potential benefits of digitalizing treatment delivery, low treatment adherence, defined as the failure to complete the full intervention (4), is a hallmark feature of DHIs that threatens to undermine their potential contribution to healthcare delivery. A systematic review and meta-analysis investigating adherence and attrition across 70 randomized controlled trials testing smartphone-app interventions found that only 34% of users completed the full intervention (1). This pattern of findings remained consistent with observations of DHI usage in natural settings (i.e., real-world environments), as industry market data show that almost 75% of users stopped interacting with their health apps after only 10 logins (5). This is problematic because it indicates that most users are not completing the full intervention as recommended, diminishing their potential to maximize positive treatment outcomes, and limiting public health impact.

The emerging research implicates poor user engagement, defined as an individual's usage, interaction, and involvement with a DHI (6), as a likely factor underlying low adherence with self-guided DHIs (1, 7, 8). With the growing evidence base linking user engagement to improved post-treatment mental health outcomes (9, 10), increased efforts have been directed towards understanding the factors influencing people's adherence and engagement with DHIs (1, 4, 11). From this research, a number of user-related (e.g., demographics and psychological) and system-related characteristics (e.g., intervention provider and features) have been proposed as potential predictors of user engagement. Particularly, studies using web-based interventions found that age, gender (12–14), education level (13, 15), and employment status (15) were linked to engagement behaviors. Other user-specific factors that have also been connected to engagement are mental health symptom severity (15, 16), motivation (4, 6, 13), self-efficacy (17), treatment expectations (4), and desire for anonymity (18). As for system and design-related factors, intervention format (4), method of content delivery (e.g., interactive vs. static platform) (19), tools for self-monitoring and goal setting (20), technology-based reminders or prompts (21) have been suggested to impact user retention (19).

With respect to app-based interventions, prior studies have found a similar range of predictors; identifying age (22), education level (23), body mass index (24), motivation (25), perceived usefulness of the app (26), and self-efficacy (27) as user characteristics related to engagement. Whereas for intervention-specific factors, in-app functions that facilitate behaviors like goal setting (28) and self-monitoring (26) have been suggested to enhance engagement outcomes. Moreover, the visual design and usability (29) of the app interface, as well as the intervention type (e.g., acceptance-based therapy) were also linked to adherence rates (1). Initially these findings appeared promising, but recent empirical and meta-analytic findings revealed that the individual and combined effects of these predictors are weak and inconsistent across studies (1, 30). As such, there is need for the field to broaden its exploration of the potential factors and processes driving people's engagement with self-guided DHIs.

Enhancing our understanding of what drives engagement with DHIs is critical for realizing their potential as a key source of therapeutic support where traditional, physically located treatment solutions are not possible or undesirable. While, in the first instance, theoretical models help to explain behavior, ultimately, they may also signal ways to enhance design of DHIs to improve behavioral goals (i.e., engagement), and may also change expectations about the therapeutic value and best approach for utilizing DHIs for treatment.

Thus, we conducted a narrative review which firstly, outlines existing theoretical positions commonly taken in digital health research, and discuss their limitations for understanding user engagement. We then propose a novel lens through which we can view and reconceptualize engagement in the digital health context using relevant—and complementary—theories of self-regulated learning (SRL) from education research. To achieve this, we reviewed five prominent models of SRL and distilled key themes (or lessons) relevant for advancing digital health research by discussing how these themes deviate from current theory and practice. Finally, we integrate these insights into a new theoretical model of engagement for DHIs, discuss potential implications for practice, and outline recommendations for future directions in the field.

## 2 Limitations to existing theoretical frameworks of digital health engagement

Concurrently, there are several existing frameworks that seek to define the likely factors shaping engagement specifically with DHIs. Most prominent are the conceptual models by Ritterband (31), Short (32), Perski (11) and their respective colleagues. Consistent with the empirical research, all three models have emphasized the importance of the system's design and delivery platform (e.g., intervention content, aesthetics, and available functions), along with the individual's unique set of characteristics (e.g., demographics, motivation, self-efficacy, symptom severity, beliefs, and treatment expectations). These models also hypothesize that people's environments (e.g., social, cultural, and physical) can impact how much they adhere to and use an intervention. However, a key limitation common to these models is that they have not sufficiently considered how the individual engages with the treatment content across the user journey. In other words, the inherent temporal element that characterizes engagement has not been made explicitly clear in these existing frameworks. While this may be indirectly implied since both Short et al. (32) and Perski et al. (11) have drawn upon theories in information technology that have considered temporality (33), engagement in both models is still depicted as a single or “absolute” event, rather than a dynamic process with varying levels of intensities. Cognizant of this underexplored concept for the digital health context, we make the case that adopting a new theoretical lens can offer important insights that can change first, how DHIs are designed, and second, how people use self-guided mental health programs, thereby enabling more practical benefits from DHIs.

## 3 The user-as-learner perspective: A new theoretical lens to view engagement

To flesh out a user journey focus for DHI engagement, we draw upon a *user-as-learner* perspective: the broad reconceptualization of the digital health user as a learner. Understanding of how people learn is central to other disciplines, such as educational psychology. The education literature has been able to characterize individual differences in learning capacity, broadly conceived as a current—though not necessarily fixed—state of ability to take in information. Most importantly, this rich body of research has developed empirically supported theories that explain how people engage with a learning task over time, why individuals differ in their learning capacity, and what to do to strengthen this capacity when needed (34, 35).

The user-as-learner perspective can also be of relevance to understanding and enhancing engagement in DHIs given the implied—yet seldom tested—assumption that individuals who enroll in DHIs have the requisite learning skills to take in and apply the information that DHIs impart. Additionally, the path to progress in DHI engagement requires acquisition of new information and learning of new skills (often to be deployed in relation to mental health concerns). Overwhelmingly, DHIs are self-guided by design, meaning that people are required to independently complete the intervention program without any external assistance, as opposed to “guided” interventions where user progress and engagement are supported by a human facilitator (e.g., health professionals) (36). As such, the majority of DHIs deliver therapeutic content using unguided lessons or modules in the form of video tutorials, audio recordings, and written text (37, 38). It is therefore essential for digital health users to (a) direct their own learning of the therapeutic information, and (b) independently manage their treatment progress (4, 37). Theoretically, this should lead to enhanced knowledge surrounding the nature of one's mental health problems and the acquisition of new behavioral, cognitive, or social skills that can be enacted to manage their symptoms. However, self-guided programs can often be overwhelming for the everyday consumer as the accountability and control of the intervention progress has shifted from the clinician to the individual—often isolated—user.

While there is limited research that applies the user-as-learner perspective to the digital health context, there is evidence, albeit indirect, suggesting that various outcomes of learning, namely knowledge acquisition and skill enactment (also termed treatment receipt and enactment respectively), are potentially relevant to treatment engagement. This is the extent to which users understand and apply the skills taught in DHIs (39). Gaining insight into the processes underlying their condition and learning about the purpose behind applying intervention skills, are believed to empower individuals to actively engage in and direct their recovery process (38, 40). Conversely, poor comprehension of the intervention material can increase the risk of users losing confidence and the motivation to adhere to their treatment plan (41). Though largely untested and inadequately fleshed out, aspects of learning have been subsumed in a number of conceptual frameworks exploring treatment engagement in both face-to-face and DHIs (11, 31, 42, 43). For instance, in Perski and colleagues' framework (11), knowledge acquisition and skill enactment are viewed as potential mediators of engagement and intervention effectiveness. Comparably, Ritterband et al. (31) also proposes these outcomes of learning as facilitators of health behavior change and eventual symptom improvement. Nevertheless, not sufficiently considered is how these facets of learning may also drive engagement itself. With the emerging research suggesting that various outcomes of learning (e.g., applying intervention skills in their day-to-day lives) may be more indicative measures of effective engagement with DHIs (28, 44), a stronger theoretical understanding of how these concepts are related is needed.

### 3.1 Learner-centric frameworks for digital health

Despite the importance of knowledge acquisition and skill enactment in psychological interventions, the digital health literature seldom mentions a guiding framework that positions the user as a learner. One potential reason for this could be the field's limited efforts to integrate findings from seemingly unrelated disciplines (11, 33). A useful starting point for bridging this gap may involve drawing insights from a different line of research, as there exists an array of learner-centric models in the education literature that could potentially be applied to explore user engagement in the digital health context. Self-regulated learning (SRL) is a key conceptual framework in educational psychology developed to understand how individuals engage with learning tasks in a self-directed way. The term SRL refers to the self-driven process whereby learners actively control their emotions, cognitions, and behaviors, using strategies oriented towards learning and, ultimately, goal attainment (45). An important assumption in SRL theory is learner agency; the recognition that individuals have the *potential* to actively control their cognitions, motivation, and behaviors throughout the process of learning. However, not everyone can or will control these to the same degree (46). This is supported by a large empirical base suggesting that people significantly differ in their SRL profiles, even among those who self-select into programs (e.g., online university courses, massive open online courses) that require considerable independent learning skills (47–50). Given their self-guided nature and focus on knowledge transfer within these self-directed learning opportunities, it is plausible that this pattern of findings may also apply to DHIs. More specifically, it may be that variability in engagement behaviors with DHIs, at least in part, reflect individual differences in SRL ability across users. This is because not all users will have the same proficiency to cope with the information imparted in a self-guided intervention program.

Interestingly, there is a growing body of research showing that aspects of SRL (e.g., self-observation, and goal-setting) have been considered in the design of commercial smartwatches and smart home systems with respect to their role in supporting increased awareness and self-management of health-related issues (51–53). Smartwatch features that enable the collection of individual health data (e.g., heart rate variability, daily step count, sleep data) indirectly supports principles of SRL, as access to this information can empower people to set personal goals, track their progress, and regulate their behaviors to improve their ongoing health. Similarly, smart home technology has drawn considerable interest in recent years for its potential to facilitate self-management behaviors in individuals with cardiovascular diseases (53, 54). The emerging research shows that smart home ecosystems can support people with monitoring their symptoms, adhering to treatment regimens, and generating feedback for self-regulation. These features enable patients to play a more active role in their recovery, which has been reported to enhance treatment compliance (55). This literature further highlights the likely relevance of the SRL framework to guide the exploration of potential facilitators of engagement in both web and app-based interventions.

## 4 Review of theoretical models of self-regulated learning

There are a number of SRL models—each differing in their points of emphasis—that could offer new perspectives for guiding research on user engagement. However, given that this will be the first attempt at incorporating SRL into digital health, rather than limiting to one specific model, we sought to investigate the potential relevance of common SRL models in the education literature. This is a practical way forward to explore the user-as-learner perspective because the consideration of SRL models may inform a new list of possible drivers of user engagement, and subsequently, viable targets for retention strategies in future DHIs. Considering the field's limited and heavily siloed understanding of user engagement, gaining new theoretical insights may be instrumental for increasing the impact of DHIs for those who opt for online treatment modalities (42).

### 4.1 Zimmerman: Cyclical Phases Model

Zimmerman's SRL model (56) is one of the most prominent frameworks in the education literature (57). Heavily influenced by Bandura's social cognitive theory (58), Zimmerman's model describes how metacognitive and motivational processes cyclically influence SRL across three interdependent phases: (a) forethought, (b) performance, and (c) self-reflection. In the current version of the model, learners in the forethought phase analyze learning tasks using skills like goal setting and strategic planning, which is facilitated and fueled by personal motivational beliefs. During performance, learners use self-control strategies such as time management and self-instruction to maintain the cognitive engagement required to complete the learning activity. During this phase, self-regulated learners also track their progress using self-observation strategies like self-monitoring and self-recording (59). In the self-reflection phase, learners appraise their recent performance and find reasons for personal successes and failures. The model posits that future task performance is shaped by a learner's self-reaction and judgments, as this can positively or negatively impact future self-efficacy and learning efforts (57, 60).

### 4.2 Pintrich: General Framework for SRL

Pintrich's model (61) of SRL is another leading conceptual framework in the education literature embedded within the socio-cognitive theory (46). The model classifies SRL into four interactive phases: (a) forethought/planning/activation, (b) monitoring, (c) control, and (d) reaction/reflection. Each phase has four areas for regulation (cognition, motivation, behavior, context), thus giving rise to a vast number of possible SRL processes (60, 62). While the different SRL phases appear to reflect a linearly ordered sequence, it is recognized that earlier phases may not always occur before later phases. Instead, planning, monitoring, controlling, and self-reflection are viewed as ongoing dynamic processes that can simultaneously occur as a learner engages and progresses with a learning task (61). The overarching theme forming the focus of Pintrich's model is the strong integration of motivational processes (e.g., goal orientations and self-efficacy) across each phase, despite its position as an independent area for regulation (62, 63).

### 4.3 Boekaerts: Dual Processing Model

Boekaerts' (64) Dual Processing Model (previously called the Model of Adaptive Learning) highlights emotion regulation as an essential aspect of SRL, as emotions can influence when, how, and why students self-regulate their learning (65, 66). The model discerns three purposes for self-regulation during learning: (a) to develop one's skills and knowledge, (b) protecting the ego from harm and threat, and (c) protecting one's devotion to the learning task (60). In the model, Boekaerts explains that learners activate the growth/mastery pathway when a learning task aligns with personal goals and positive emotions are triggered. This encourages learners to commit and metacognitively sustain their engagement to the learning task without needing much extrinsic assistance because the learner is intrinsically driven to develop their competence (64). Conversely, learners initiate the well-being pathway when tasks are perceived as difficult or threatening to the ego and negative emotions are triggered. When the well-being pathway is activated, students are motivated to protect the ego from harm by either avoiding or giving up the task to prevent further unpleasant emotions (64, 65). Changes in appraisal may also happen, whereby students initially activate the growth pathway but redirect themselves to the well-being pathway after experiencing negative triggers or cues for failure, likely due to previous failed attempts. However, students may also revert back to the growth pathway by implementing volitional strategies to re-focus on the learning task at hand (65).

### 4.4 Winne and Hadwin: Metacognitive Model SRL

Shaped by the Information Processing Theory (IPT), Winne and Hadwin's model offers a metacognitive conceptualization of SRL (67). The authors postulate that SRL consists of four recursive and loosely sequenced phases: (a) task definition, where the learner generates their perceptions of the task; (b) goal setting and planning, where the learner forges goals and formulates a plan to achieve them; (c) enacting study tactics and strategies, where the learner executes the plan; and (d) metacognitive adapting, where the learner adjusts their strategies and future approach based on their recent task execution (68). Five IPT-influenced processes (referred to using the COPES acronym) are proposed to underpin each phase: conditions, operations, products, evaluations, and standards (67). *Conditions* are the resources accessible by the learner and constraints specific to the task or environment (e.g., instructions, time). *Operations* are the various SMART cognitive strategies learners use to manipulate and process information, this includes searching, monitoring, assembling, rehearsing, and translating (60, 69, 70). *Products* are the outcomes (i.e., information) generated by the aforementioned SMART operations. *Evaluations* refer to the internal feedback generated from self-monitoring concerning the discrepancy between the products and the *Standards;* the set of criteria the learner considers to be the optimal outcome (63, 67).

### 4.5 Efklides: Metacognitive Affective Self-regulated Learning (MASRL) Model

Building on the ideas proposed in earlier SRL models (e.g., Zimmerman, Pintrich, and Winne and Hadwin), Efklides' MASRL model focuses on the regulation of motivation, affect, and cognition, and explains their interrelated yet distinct roles throughout the SRL process (71, 72). The model proposes two levels of SRL functioning. First, the *Person-level* (i.e., macro-level) depicts the top-down processes in SRL and shows how a learner's goals and personal traits (e.g., self-concept, motivation, affect etc.) can interact with each other to impact decisions relating to engagement with a learning task (72). Conversely, the *Task x Person-level* (i.e., micro-level) represents the bottom-up regulation of learning where the processes are less characterized by the learner's traits and more by the specific demands of the learning task. This level is more data-driven in nature, as metacognitive monitoring of task processing and performance becomes the driving force behind the learner's actions. The two levels of SRL functioning are theorized to interact and reciprocally inform each other to influence the learner's affect, motivation, and effort regulation in a specific task.

## 5 Key themes in self-regulated learning theory: Lessons for digital health research

As articulated in Section 3, digital health interventions (DHIs) have an underlying—and often implicit—emphasis on knowledge acquisition and psychoeducation, even if the stated primary goal is symptom improvement. Here, we seek to give greater prominence to this underlying dimension of learning as essential to treatment engagement and positive outcomes, by connecting DHIs to key principles of learning articulated in education research. Therefore, we propose the *user-as-learner* perspective as a novel approach for understanding individual differences in engagement with self-guided DHIs. Learner-centric models, particularly self-regulated learning (SRL), offer a well-established conceptual framework through which we can re-appraise the self-directed learning journey of individuals engaging with DHIs.

Wholistic evaluation of five models of self-regulated learning (56, 61, 64, 68, 72) revealed three key themes to guide a user-as-learner perspective for DHIs: (a) common drivers of engagement in SRL, (b) the temporal nature of engagement and its drivers, and (c) individuals may differ in learning capability. In the following section, each theme will be discussed according to its potential for extending the current understanding of user engagement with DHIs. Finally, to integrate these findings into the broader literature, we propose a new model of engagement for the digital health user journey and draw out important clinical and practical implications and directions for future research to leverage key insights from SRL for DHIs.

### 5.1 Theme 1: Common drivers of engagement in SRL

SRL models offer a cohesive overview of the key factors underpinning the process of engagement in the learning context (broadly conceived). We use the term “broadly” to reflect the variation in nomenclature used by different SRL researchers to describe conceptually similar constructs (e.g., self-efficacy and confidence). Through appraising the five SRL models, we deduced a list of recurring factors or skills that have been theorized to influence people's engagement with learning tasks (see Table 1). Whereas some SRL factors are common across all models (e.g., motivation), others are more unique to specific frameworks, due to the slight differences in factor emphasized by each author (63, 72). Nevertheless, there is a general consensus that higher levels across these SRL variables can facilitate and sustain effortful engagement throughout one's learning journey, and in turn, promote positive learning outcomes (56, 61, 64, 68, 72). Thus, we henceforth refer to these variables collectively as “drivers” of engagement. Conversely, lower levels across these drivers are viewed as potential threats to engagement and can hinder the learning process.

**Table 1:** Broadly recurring SRL drivers across prominent models of SRL.

SRL driver	Zimmerman	Pintrich	Boekaerts	Winne & Hadwin	Efklides	DHI literature
Psychological						
Motivation	✓	✓	✓	✓	✓	✓
Self-efficacy	✓	✓	✓	✓	✓	✓
Task interest	✓	✓	✓*	✓*	✓*	Hypothesized
Task value	✓	✓	✓	X	✓*	Hypothesized
Outcome expectations	✓	✓*	✓	✓*	✓	✓
Self-control	✓	✓*	X	✓*	✓*	Insufficiently addressed
(Positive) emotions	✓*	✓*	✓	✓*	✓	Hypothesized
Behavioral						
Goal setting	✓	✓	✓	✓	✓*	✓
Metacognition	✓	✓	✓	✓	✓	Insufficiently addressed
Social						
Social factors	✓	✓	✓*	✓	X	✓

✓ = explicitly addressed; ✓* = indirectly addressed; X = not addressed.

As summarized in Table 1, several key SRL drivers have already been considered in digital health research, both in empirical studies looking at predictors of user engagement, and in existing theories of digital health. For instance, five of these drivers including motivation, self-efficacy, outcome (treatment) expectations, social support, and goal setting have been associated with positive engagement outcomes in a number of DHI studies (4, 6, 13, 17, 27). Moreover, drivers like task interest, (positive) emotions, and task value have been hypothesized as potential facilitators or attributes of engagement in various conceptual frameworks of DHIs (11, 31, 32), albeit lack the evidence base to confirm their influence on DHI usage. Despite the potential overlap in variables explored across these disciplines, a key point of difference is how some of these drivers are conceptualized and studied with respect to their potential role in influencing engagement behaviors. This is evident in drivers like metacognition and self-control, which have seldom been addressed or sufficiently considered in digital health research, though their closest comparators are concepts such as self-monitoring, self-evaluation, and goal setting.

SRL researchers describe learners as *metacognitively* active when they effortfully observe, reflect, and modify their behaviors to improve learning performance and, through this process, may be expected to observe positive progress towards their goals (68). Superficially, this may seem comparable to self-monitoring and goal setting as understood within the digital health literature, however there are important—though complementary—points of differences. In the DHI context, users are typically encouraged to learn about the antecedents and consequences of problematic behaviors in order to intervene earlier in the onset of these problematic behaviors (73). The learning emphasized in this DHI context is about monitoring symptoms, whereas in SRL, the focus is on monitoring one's *learning*, a higher order cognitive task which may inform whether the user has appropriately understood the treatment content and can apply it effectively within their daily lives. More specifically, the SRL focus is about being or becoming metacognitively aware of one's progress in acquiring and enacting new knowledge and skills from a learning task.

Adopting the SRL perspective would allow us to apply a metacognitive lens to how key skills are utilized in DHIs, by also asking individuals to reflect on their comprehension and attempted implementation of the content received. Quizzes and self-assessment may be deployed to evaluate learning in DHIs (22), yet SRL approaches would go a step further in offering learning strategies to enhance understanding in cases where knowledge and/or enactment gaps are evident. More specifically, digital health users would be encouraged to reflect on whether they (a) understood the intervention module they have just engaged with, (b) felt confident with putting the newly acquired skills into practice, and (c) getting them to reflect on their attempts at applying these skills. This focus on self-awareness is central to understanding one's strengths and weaknesses with learning intervention skills and could help users identify which modules require further engagement to facilitate their primary goal of symptom improvement.

Similarly, self-control is another variable mentioned in both bodies of research, although with notably different foci. In the SRL literature, self-control has been viewed as an individual's discipline to engage in and sustain goal-directed behavior (74, 75). This is an effortful process that requires a set of self-management strategies (e.g., time-management, self-instruction, and self-consequences) to help the learner maintain concentration and interest with their learning tasks (56, 57). On the other hand, self-control in DHIs is typically about minimizing harmful behaviors, often in-the-moment. For example, DHIs may focus on identifying when an urge is present and learning how to control and override temptations. Integrating SRL theory to how common drivers are understood in the digital health literature would encourage the field to explore the role of self-control in a new angle. In particular, investigating its impact on successful learning and completion of intervention content. Though seldom acknowledged from this perspective, exercising self-control (and effective time management) to learn the information received is relevant, and likely essential, for treatment engagement. This is because individuals with greater self-discipline are theoretically more inclined to use the necessary strategies that will help sustain their motivation to learn successive intervention content.

In addition to these nuances in how specific concepts are understood, the interrelations between certain constructs are more strongly articulated in one discipline than the other. For instance, motivation in digital health is often referred to as an individual's readiness to change, or intention to engage in and complete treatment (76, 77). However, SRL models suggest that this concept is more complex, and characterize motivation as a dimension of self-regulation influenced by multiple other drivers (e.g., self-efficacy beliefs, goal-directed behaviors, and task interest) throughout the learning process (35, 78). Thus, adopting the user-as-learner perspective may bring more attention to the inter-relations between multiple drivers and highlight possible mediating or moderating pathways for increased engagement with DHIs (further discussed in Theme 2).

Hence, the two lessons from Theme 1 are as follows. First, there are a number of potentially relevant SRL drivers that have been insufficiently considered or directly tested in the digital health context. This, therefore, suggests the need for broadening the range of predictors that we measure in studies investigating engagement with DHIs. Second, we should also think about appraising and conceptualizing these drivers in a new light. That is, rather than limiting our understanding of these constructs to the DHI context, we should consider how they are defined in different disciplines, particularly in bodies of work with theoretical models that can more comprehensively explain the interrelations between key drivers. A greater understanding of the potential interactions and mediating pathways can suggest new opportunities for intervention to enhance user engagement and guide the prioritization of what to intervene upon.

### 5.2 Theme 2: The temporal nature of engagement and its drivers

The second key theme across the SRL models is the notion that learning is an evolving and dynamic process (79). That is, the constructs that drive engagement are not constant, and instead can wax and wane over time. In turn, increases or decreases in these drivers can also affect levels of learner engagement. Using Boekaert's model (64) as an example; a student's appraisal for a learning task can change when there is a shift in emotional experience during task performance. For instance, challenging tasks initially perceived as easy may elicit feelings of hopelessness after several failed attempts. However, the student can also re-appraise the situation using emotion regulation strategies to deescalate those negative emotions that hinder effort investment for the learning task (64, 65). Two of the five SRL models (56, 68) also suggest that engagement itself may provide a feedback loop for the drivers by enhancing motivation when one feels growing competence from successful task completion, or diminishing motivation in response to difficulties or failures. In broader terms, the outcome of engagement may also have an effect on its drivers, which in turn, can reinforce more or less of this positive outcome. This indicates that there are complex interactions occurring between different SRL variables over time (72), which can determine the degree to which an individual seeks to initiate successive cycles of learning (59).

This theme offers three important lessons with notable implications for the digital health context. The first, is that if these drivers of engagement do fluctuate over time as SRL research suggests, then *when* we measure them in the user journey matters. To date, much of the empirical work in the digital health field investigating predictors of engagement have been largely limited to baseline data, particularly in studies measuring drivers like motivation and self-efficacy (6, 13, 27, 76, 77). This is despite the large evidence base from related disciplines showing that motivation for treatment and behavior change can vary considerably over time (80–84). Relying on baseline predictors alone may be a critical oversight, as new users can often present with an inflated attitude during the onboarding phase. Their initial motivation, however, may diminish when faced with unexpected challenges (e.g., difficulties understanding psychoeducational content), and thus, may not reliably predict engagement patterns later in the user journey. Therefore, it is essential to recognize that those factors driving initial uptake may differ to those factors sustaining engagement and eventual symptom improvement. An important takeaway for digital health researchers is to consider measuring these drivers not only at baseline, but also *during* the intervention. Exploring the relative contributors of engagement at different milestones in the user journey would serve to test this possibility if certain drivers are more important at particular times, and if time of measurement impact predictive ability.

The second point of relevance for digital health research is that we need to better understand the complex relationships between the different drivers and engagement itself. As briefly discussed in Theme 1, there are potential moderating and mediating pathways that need to be considered. Ignoring how the different variables inter-relate more broadly (e.g., looking at one variable alone without the interaction of the moderator) can result in an inaccurate estimate of the true effect of each driver on engagement. To articulate the complex interplay between different variables, we describe the potential impact of self-efficacy and its proposed correlates on digital health engagement. A user's self-efficacy beliefs about their ability to learn new intervention skills can determine not only their motivation, but also their goal setting behaviors, both of which can influence one's efforts to self-regulate their engagement with the intervention task (78). Furthermore, this engagement or SRL of content received (dependent on the user's initial experiences) can form a positive or negative feedback loop that sustains or diminishes their ongoing self-confidence and motivation to persist with successive treatment content. Thus, to advance our understanding of people's likelihood of engagement, it is imperative to build upon existing models of digital health, to better define these complex—and potentially bi-directional—relationships between different variables.

Finally, if we can understand the temporality of engagement and its drivers, as well as how they relate to each other throughout the user journey, we can identify more opportunities to intervene. Importantly, this could also highlight the need for different intervention approaches at different stages to impact specific levers and bolster treatment engagement. To put it simply, digital health developers should consider implementing targeted interventions at specific points throughout the self-guided program to enhance key drivers of engagement where necessary. Efforts to increase engagement across the user journey could support users, particularly those with lower SRL skills, to complete the recommended psychoeducational modules and facilitate symptom improvement.

### 5.3 Theme 3: Individuals may differ in learning capability

The third key theme emerging from these common models of SRL is the recognition that people significantly differ in their capability for SRL, but also emphasizing that this capability for SRL skills is malleable and teachable. Learners are viewed as active participants in their learning journey (46, 56, 61, 65, 68, 72), meaning that with effortful attention and action, SRL skills can be developed and improved over time. To ground this discussion, there is a rich evidence base in the education literature showing that people substantially differ in their SRL profiles, particularly in online learning environments (47–50). Despite being cognizant of the general academic skills (e.g., time management, planning, motivation) required to successfully complete a self-guided syllabus, the consistent pattern across many online courses is low completion and high dropout rates (50). This may, at least in part, reflect the wide range of SRL capacities of people who self-select into digital learning environments, showing that not all learners have the ability or skillset to successfully complete an online course with minimal guidance.

Consideration of these individual differences in people's SRL capacity is potentially relevant in the digital health context because not all users have the required abilities or skillset at time of commencement. Nonetheless, there is an ongoing and misguided assumption that those who sign up for DHIs will know how to effectively manage and direct their learning of the content received. As a result, there is typically no direct attempt to (a) assess people's current SRL capacity, and (b) enhance these skills where necessary. For these reasons, many DHIs are not well-equipped or designed to support users who start out with low levels of SRL. This raises serious practical implications for those relying on DHIs as their primary mode of treatment (due to geographical, financial, or time restraints), as many first-time users may find intervention tasks difficult or overwhelming without the support of a clinician. Difficulties engaging with the treatment content may in turn, diminish people's motivation to persist in their user journey and complete the full program as recommended. Using SRL as a guiding framework, we believe it may be essential for those who enroll into DHIs to (a) be aware of their current learning abilities, and (b) effortfully enhance their SRL skills to drive ongoing engagement with the intervention material. Integrating an SRL perspective into the design phase may open up new avenues to overcome issues with sustained engagement. For instance, the development of support material to teach users the necessary skills to complete their DHIs and relieve unwanted symptoms.

## 6 Proposing the self-regulated learning model of digital health intervention

These themes drawn from SRL research offer complementary elements to enhance understanding of engagement throughout the DHI user journey. Existing theoretical models of engagement (11, 31, 32) in digital health have emphasized the importance of intervention design and considered a range of potential drivers of engagement and successful outcomes. This includes user-related factors such as demographic characteristics, motivation, self-efficacy, and symptom severity, as well as system-related factors like interface design, usability, visual appeal, method of delivery, and available functions. However, a common limitation across these models is that they do not consider how the individual engages with the intervention *throughout* the user journey, which implicitly has a temporal element. This limited emphasis on the temporal aspect of the user journey has potential to miss opportunities for developing retention strategies that can mitigate risks of dropout at specific stages. The SRL models identified in Section 3, on the other hand, inherently tap into this temporal dimension of engagement, which encourages further thought about how to structure the learning experience, what to measure across the user journey to evaluate success or need for additional support, and to also consider what may drive engagement at different stages of the journey.

**Figure 1:**
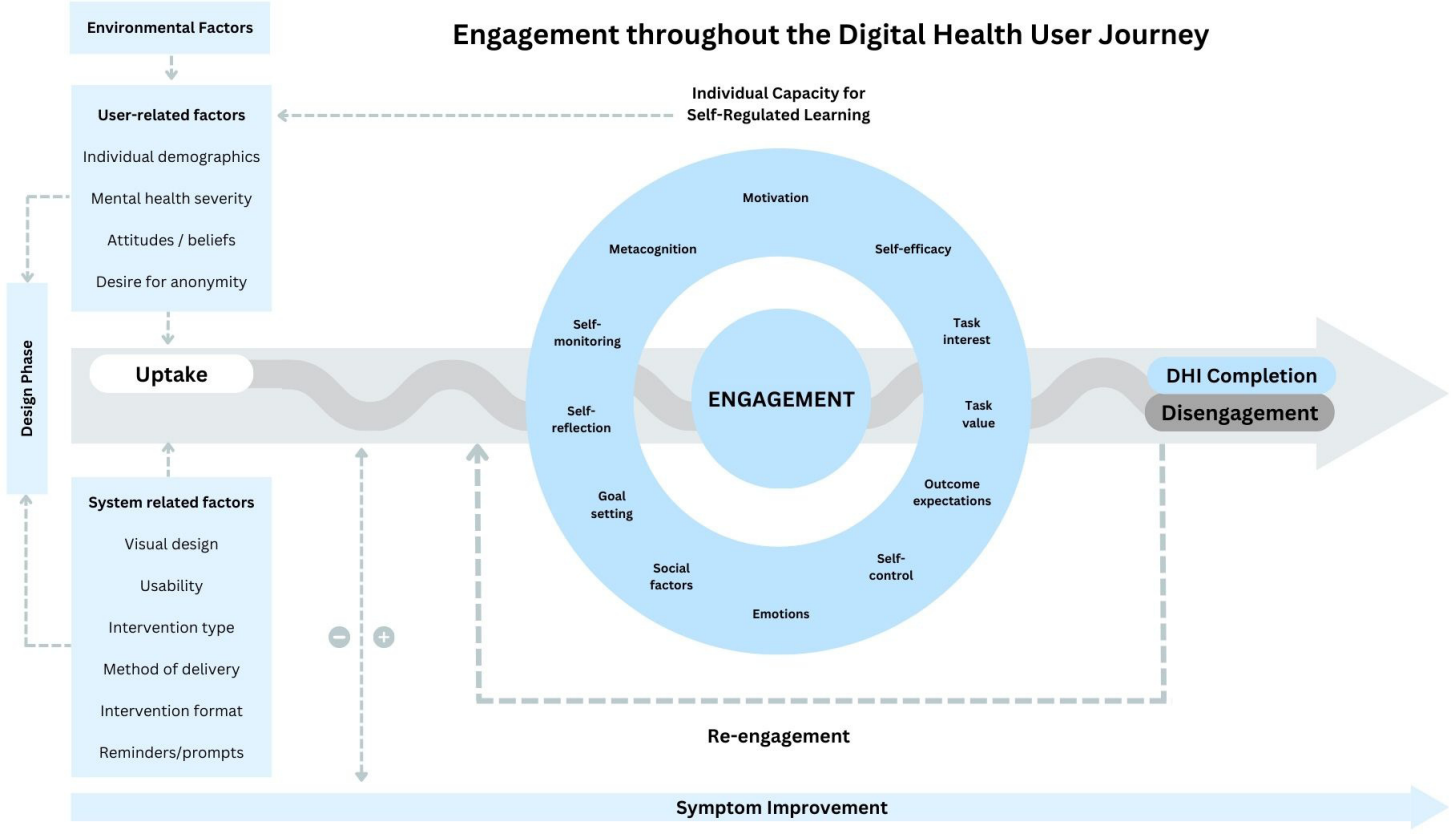
SRL model of DHI engagement.

Therefore, to consolidate the theoretical perspectives from SRL models into these earlier frameworks (11, 31, 32), we propose a new model (see Figure 1). Our model aims to explicitly characterize the complex and temporal nature of engagement throughout the DHI user journey and the key drivers underpinning this process. Whereas some factors are most relevant during the design phase of the intervention, others may be drivers of initial uptake or continued use. A salient feature of our model is the notion that the SRL drivers facilitating engagement are in fact *fluid* and likely interact with each other over time in feedback loops that may signal positive progress, or risk of disengagement and drop-out. Likewise, engagement itself plays a role in sustaining or diminishing the ongoing level of its key drivers. This means that some factors will remain relevant as the individual continues to use the DHI, so it is vitally important that we consistently monitor how these variables evolve across the user journey, rather than simply taking a single snapshot of these at baseline (i.e., prior to commencement of an intervention). The proposed model also depicts the bidirectional relationship between engagement and symptom improvement, demonstrating that level of symptom improvement can drive further engagement, just as engagement may also lead to greater symptom improvement.

## 7 Potential implications of adopting the SRL model of DHI engagement

The proposed model (Figure 1) was designed to address the limitations of the current research on DHI engagement highlighted in Section 2. If this revised framework can improve how the field understands and predicts people's use of DHIs over time, it carries potential implications for enhancing theoretical knowledge, optimizing DHI design, and enhancing the implementation of DHIs in practice.

With respect to potential advances in knowledge, having a comprehensive framework that integrates SRL theory to understand engagement expands the current list of potential factors that are theorized—and, over time, may be empirically demonstrated—to drive or hinder this process. This has direct relevance for strengthening our predictive ability of who may disengage from DHIs prematurely. In addition, the model's emphasis on the temporality of engagement can provide deeper insights into (a) critical points along the user journey where engagement is likely to drop off, and (b) the key drivers that are most influential at each stage of the user journey. The temporal element also articulates the importance of extending beyond the all-too-common practice of using baseline predictors to assess engagement and treatment outcomes in DHI studies. While some authors have previously raised the importance of considering temporality in prediction of engagement (30, 85–87), our model provides a more fully formed vision of what impacts engagement and when, which should better guide study and DHI design.

These insights not only advance the theoretical understanding of engagement but also have meaningful implications for practice and future design of DHIs. The proposed model appreciates that individuals with high SRL abilities can benefit from DHIs immediately, whilst also acknowledging that in reality, not everybody signing up to DHIs will have these requisite skills. Thus, in practice, individuals interested in using DHIs may benefit from completing a screening tool that can assess their SRL capacity to determine their suitability for self-guided treatment approaches. Screening during the onboarding phase could help experts identify individuals who may struggle with self-guided treatment content, enabling opportunities for targeted support that can enhance their SRL skills. It may be that those with lower SRL need face-to-face contact with health professionals for a brief pre-training session on how to maximize positive outcomes from self-guided interventions, or perhaps building supplementary features into DHIs to target specific SRL deficiencies (e.g., goal setting tool to help the end-user set appropriate and personalized treatment goals). Pre-screening can also lead to more optimized resource allocation across mental healthcare service providers by supporting clinicians in matching patients to the treatment modality that best aligns with not only their symptom profile but their SRL capacity. In turn, this allows the more intensive measures (e.g., in-person therapy) to be prioritized for individuals with little to no SRL abilities.

Concurrently if the proposed model is accurate in suggesting that (a) SRL is indeed a key ingredient for DHI engagement, and (b) the significance of each driver changes across the user journey, it has the potential to transform and shape how future DHIs are designed. This may involve designing DHIs in such a way to ensure that the self-guided content is structured to scaffold the user's learning of psychoeducational information or scheduling regular activities within the DHI program that encourage users to engage in SRL behaviors, such as self-reflection. For smartphone app interventions, this may involve using pushed notifications that prompt users to complete weekly self-reflection tasks in relation to their progress with learning and applying the treatment content. DHI developers may consider embedding brief assessments (e.g., quizzes) throughout the user journey to evaluate whether users understand and implement the treatment content correctly, and if not, provide touchpoints with experts or clinicians to correct mislearnings.

## 8 Recommendations for future directions

In light of these potential implications, we have derived five actionable recommendations for future efforts in the field. The first two recommendations focus on addressing current knowledge gaps, while the third is centered around improving DHI outcomes in practice. Finally, the fourth and fifth recommendations outline potential steps for enhancing future DHI design.

(i) First, more empirical evidence is needed to show that SRL is related to engagement in DHIs. A useful starting point would be expanding the range of predictors that we currently measure to include the full range of key drivers in SRL. As detailed in Theme 1, several SRL drivers have already been considered in the digital health literature, however, there are still a number of other potentially relevant drivers (e.g., metacognition, self-control, positive emotions, task interests, and task value) that have been insufficiently acknowledged or directly tested in this context. Determining the predictive power of these drivers on user engagement may also necessitate the creation or modification of SRL measures for digital health research.
(ii) Second, the digital health field should acknowledge the temporal nature of these drivers and its relation to user engagement, by increasing the granularity of measurements in future studies. Rather than limiting to baseline assessments, researchers should consider adding more time points of data collection throughout the user journey to better understand how and which drivers fluctuate over time, and how these changes may influence people's engagement. Given that users can often start with an inflated attitude during initial uptake, we anticipate that those temporal trends closer to the time of drop-out are more accurate predictors of user engagement than measurements captured at baseline.
(iii) Third, professional development opportunities for health professionals are needed to ensure they are competent in supporting individuals opting for digital health options. Digital health experts should consider developing a focused, single-session training program for clinicians to improve understanding of the self-regulated learning processes required for successful DHI completion. Such a program could include guidance on identifying and addressing barriers to SRL, strategies for facilitating sustained engagement, and techniques for supporting their clients' learning of psychoeducational content and new skills throughout the user journey, ultimately maximizing treatment outcomes.
(iv) Fourth, efforts are needed to comprehensively review and map out the current evidence base for brief interventions that can quickly and effectively bolster people's engagement with learning tasks. Where gaps exist in the literature, researchers should prioritize the development of new resources or modules that can enhance specific drivers or SRL skills (e.g., goal setting). Embedding the appropriate evidence-based resources within DHIs may be the key to supporting users in enacting important skills that help them stay engaged throughout the user journey and improve overall treatment adherence.
(v) Finally, to confirm if SRL-specific design features (e.g., self-monitoring tools) are important for enhancing outcomes with DHIs, researchers should consider comparing a range of available DHIs that include these features, vs. those that do not, and evaluate them to see if there are differences in engagement metrics, symptom improvement and treatment outcomes.

## 9 Conclusion

This paper was the first to evaluate the potential relevance of SRL theory to the digital health context. From the five SRL models included in this review, three key themes were identified and applied to extend our understanding of engagement with DHIs, given their underlying emphasis on knowledge acquisition and psychoeducation. The three themes discussed were (a) how SRL models can provide a complementary lens for exploring drivers of engagement, (b) that engagement and its drivers temporally fluctuate, and (c) the need to consider individual differences for learning capacity in those who sign up to DHIs. As such, we make the case that reconceptualizing the digital health user as a *learner* can invite new theoretical perspectives for improving predictive and conceptual models of engagement throughout the user journey. This may inform the development and implementation of retention strategies that can better support people to engage with and complete their DHIs as recommended. Additionally, by accounting for differences in user SRL abilities, these insights can help match individuals seeking mental health support with the most appropriate treatment modality (e.g., self-guided, blended, or in-person therapy). Ultimately, this may enable more efficient resource allocation within the healthcare system and enhance treatment outcomes on a broader scale.
